# The Doings of St. Dunstan's Men

**Published:** 1918-05-11

**Authors:** 


### THE ADVENTURES OF THE BLIND.

### The Doings of St. Dunstan's Men.

After reading from cover to cover the third annual report of St. Dunstan's Hostel for Blinded Soldiers and Sailors, we believe that its interest is as least as much due to the editing and presentation as to the interesting life with which it deals. To illustrate the severe criticism which is thus implied on the ordinary hospital report, we would ask hospital secretaries to compare their treatment of "thanks and acknowledgments" with those, hardly longer, in the St. Dunstan's report. The ordinary thanks from an abysmally dull and prosaic list of empty phrases: the notes of thanks in the St. Dunstan's report have a trick of mentioning something interesting for which the brief thanks sound natural and sincere. Apart from this technical point of interest, the report is really a survey of the work, for it deals with particulars, not generalities, with this or that piece of work for this or that person, not with results or categories. So far as the life of an institution can be conveyed by description, that of the St. Dunstan's men is conveyed here in all its versatility, with all its odd points of interest, in this or that out-of-the-way corner at home or abroad.

The first emotion excited by the institution itself is often incredulity, for the blind are perceived to be leading the blind without falling into the ditch. As a child has to teach itself to see, to focus, and to recognise objects and distances, the blind man has to be taught to sense them. This teaching is divided between the classroom, the workshop, and the companionship of teachers and pupils in the like case. The new handicap, however, imposes a severe mental strain at first. It is as exhausting to learn to be blind as to sustain for the first time a conversation in a foreign language. The two exercises are very similar, and equally fatiguing at first. While the new faculties are cultivated the old deteriorate, as a new crop will oust an old in nature, and the Braille writing is not acquired without corresponding and curious deterioration in caligraphy.

The occupations and trades open to the men fall, roughly, into two classes, those manual industries of which cobbling, mat-making, basket-making, and joinery are the chief; and the occupations which are, rather oddly, not called manual, for they include massage and typewriting, either of which, without the aid of the hand, we suppose that even a blind man would find difficult to master. There is, too, the intermediary occupation of poltry farming; and this province of the pessimist seems to be occupied with success by St. Dunstan's men, who learn its methods and use its trap-nests on the St. Dunstan's poultry farm. Indeed, their families and relatives are also trained free of charge at another farm which is run on the same principles as those of St. Dunstan's. Since details convince, we may add that the blind learn to distinguish the different breeds by touch, while trussing, by comparison, offers no difficulties. Among the masseurs none has failed to pass the stiff examination of the Incorporated Society of Trained Masseurs; and after training the men join the staffs of military hospitals or the Almeric Paget Massage Corps, and earn salaries of £2 10s. a week or more, besides special commendation. The typewriter which now enables a condensed Braille to be transcribed as rapidly as the script of an ordinary shorthand writer has led many men to resume their old places in offices, or to start more efficiently than before in new posts. Finally, telephone operating is taught. This opens posts in big offices to the blind, for in the office exchanges the drop-shutter system exists, and though each shutter looks identical, in a few weeks the blind learn to hear which one has fallen.

But commercial occupations are not the only things taught. Dancing, swimming, rowing, chess, and cards, these, too, are learnt. It is almost a surprise to learn that the cox of the rowing boats has to be troubled with eyes, or that in the dancing which goes on the lady who is steered so carefully herself can see. Almost everyone plays an instrument, and the debating society is still in session.

St. Dunstan's and its annexes numbered in March 578 men, and the accommodation is being increased for eighty more. For those whose loss of sight is not their only loss there is a home for their permanent care at Ilkley, in Yorkshire, and Scotchmen have the choice of going to Newington House, Edinburgh, which is affiliated to St. Dunstan's. Of the 434 men who up to March last had passed its doors, 10 per cent., "for some reason in their health or habits, have left partially trained, untrained, or untrainable." Without this proportion of failure the successes would hardly be believed.

But on leaving St. Dunstan's a man is not left wholly to himself. He is equipped, stocked, and settled to practise the occupation he has been taught. Visits are made to him regularly; the raw material of his work is bought and supplied to him at cost price; and lastly, he is helped to place his goods on the market either locally or at a central depôt. The successes in either market are described in the interesting collection of letters from men who have thus re-entered commercial life, which fill the latter pages of this report. The letters do not seem to have been censored; but if there are some, unpublished, which mention failure, we should like to see all of them but the writer's name. Every experience is of interest in this new development of the blind. Of course the after-care arrangements must be permanent, and have been framed to endure. They are the modern parallel to the blind man's stick of old, the one necessary support and reserve of strength inside him. Of similar intention is the new fund to raise the Government allowance to 5s. for every child born to a blinded soldier. The men seem to earn by their trades £2 or £3 a week; sometimes much more. We read of the turnover of the tobacconist, the newsvendor, the cobbler, of the

**THE ADVENTURES OF THE BLIND—*(continued)*.**

masseur's lengthening list of clients, of the commercial man's rises in salary, of the poultry farmer's average of nearly 100 eggs per bird; and the lowest earnings quoted seem to be £1 9s. 1d. One former patient has even turned politician, and is one of the three soldier members of the Saskatchewan Legislature. His constituents, at least the objects of his duties, are the soldiers or discharged men of the province. No wonder he writes of the interest of his work. One is an author (of "Englishman, Kamerad"), another the editor of *St. Dunstan's Review*, another the historian of the New Zealand Expeditionary Force. The measure of success, however, is best seen in the return to this country of several blinded Colonials, who had gone to their distant homes without visiting St. Dunstan's. Having met the men trained there, these have left the Colonies again for England to receive the training which they missed.

